# Opposite Associations of Trunk and Leg Fat Depots with Plasma Ferritin Levels in Middle-Aged and Older Chinese Men and Women

**DOI:** 10.1371/journal.pone.0013316

**Published:** 2010-10-13

**Authors:** Hongyu Wu, Qibin Qi, Zhijie Yu, Liang Sun, Huaixing Li, Xu Lin

**Affiliations:** Key Laboratory of Nutrition and Metabolism, Institute for Nutritional Sciences, Shanghai Institutes for Biological Sciences, Chinese Academy of Sciences and Graduate School of the Chinese Academy of Sciences, Shanghai, China; Karolinska Institutet, Sweden

## Abstract

**Background:**

Few data have been published on the associations of ferritin with trunk and leg fat depots. We aimed to investigate these associations in a Chinese population.

**Methodology:**

Trunk fat mass and leg fat mass were determined in a cross-sectional sample of 1,150 Chinese (479 men and 671 women) aged 50–70 years by dual-energy X-ray absorptiometry scan. Fasting plasma ferritin was measured.

**Principal Findings:**

Plasma ferritin was positively correlated with waist circumference, waist-to-hip ratio, total body fat and trunk fat mass, but inversely correlated with leg fat mass in men (r = 0.16, 0.26, 0.19, 0.22 and −0.12, respectively, all *P*<0.05) and women (r = 0.16, 0.16, 0.08, 0.17 and −0.12, respectively, all *P*<0.05). Multivariate regression analysis showed that ferritin levels increased with larger trunk fat mass (β = 0.33 ± 0.08 for men and β = 0.21 ± 0.05 for women, both *P*<0.001) while decreased with larger leg fat mass (β = −0.12 ± 0.09, *P* = 0.15 for men; and β = −0.14 ± 0.05, *P* = 0.005 for women). Moreover, plasma ferritin levels decreased with increasing tertile of leg fat mass among each tertile of trunk fat mass.

**Conclusion:**

This is the first study to report the opposite associations of trunk and leg fat depots with plasma ferritin levels.

## Introduction

Elevation of circulating ferritin concentrations, reflecting body iron overload, has been well established to be associated with diabetes and its risk factors such as obesity, metabolic syndrome and chronic inflammation [1]–[5]. Several previous studies found that increased circulating ferritin levels were associated with indices of abdominal obesity such as waist-to-thigh ratio [6], waist-to-hip ratio [1] and abdominal visceral and subcutaneous fat area [2]. In contrast to the adverse effects of excess abdominal or trunk adiposity, leg fat depot has been reported to have favorable associations with glucose metabolism and other metabolic risk [7], [8]. However, until now, few data have been published on the association between ferritin and regional fat distribution, particularly leg fat depot, evaluated by whole-body dual-energy X-ray absorptiometry (DXA) scan. Therefore, we aimed to investigate the association of plasma ferritin concentrations with trunk and leg fat accumulation measured by DXA in 479 men and 671 women aged 50–70 years in shanghai, China.

## Methods

The study population was a sub-set of participants in the Nutrition and Health of Aging Population in China Project, a population based cross-sectional survey among 3,289 residents aged 50–70 years from Beijing and Shanghai in 2005. The study design and protocols have been described in detail elsewhere [9]. Briefly, information on demographic characteristics, disease status, lifestyle practice, and physical activity was collected using a standard questionnaire. Waist and hip circumferences were measured and overnight fasting blood samples were collected. Whole-body DXA scan was performed using Hologic QDR 4500 W scanner (Hologic Inc., Bedford, MA, USA). Fat mass of whole body, trunk (including thorax, abdomen and pelvis) and leg (sum of left leg and right leg) regions were analyzed with software provided by the manufacturer. Plasma ferritin was measured by using a commercially available particle-enhanced immunoturbidimetric kit (Shanghai Gensource Co., Ltd., Shanghai, China) [3]. As the DXA scanner was available only in Shanghai fieldwork, participants from Shanghai were invited to take a DEXA scan and 1179 people (71.5% of 1648 Shanghai study participants) were recruited and completed the whole body scan. Among Shanghai participants, there is no significant difference between people who attended and who did not in age, waist circumference, family history of chronic disease, smoking and alcohol drinking. Subjects with irremovable metal artefacts and/or jewellery were excluded from the present analyses. Finally, a total of 1150 participants (479 men and 671 women) with DXA scan and plasma ferritin measured were included in the current analyses. Study protocol was approved by the Institutional Review Board of the Institute for Nutritional Sciences, and written informed consents were obtained from all participants.

### Statistical analysis

Analyses were performed in men and women separately due to gender differences of fat distribution. Trunk and leg fat were normally distributed in both genders. Ferritin concentrations were natural log-transformed before analyses. Correlation coefficients between ferritin and waist circumference, hip circumference, waist-to-hip ratio, total body fat mass, trunk fat mass and leg fat mass were calculated by Spearman correlation analysis. Multivariate linear regression models were used to test the determinants of plasma ferritin concentrations. Analysis of covariance was used to compare ferritin levels according to the tertiles of trunk fat mass and leg fat mass. Potential confounders included age, residence, educational attainment, alcohol drinking, smoking, physical activity, self reported cardiovascular disease (CVD), family history of diabetes and CVD and menopause status (in women participants). Data were analyzed using Stata (version 9.2; College Station, TX). *P*<0.05 (two sided) was considered statistically significant.

## Results

The characteristics of the study participants are shown in Table 1. As expected, women have significantly higher mean levels of hip circumference (*P*<0.05), more total body, trunk and leg fat mass (all *P*<0.001), although men have significantly higher mean levels of total body mass and larger waist circumference (both *P*<0.05) and they more likely to be alcohol drinkers and smokers. Plasma ferritin concentrations were higher in men than in women (median [interquartile range]:149.8 [97.5–219.5] vs.111.6 [73.10–165.4], *P*<0.001).

**Table 1:** Characteristics of study participants*.

	Men (N = 479)	Women (N = 671)	*P*
Age (yr, n, %)	59.0±5.9	58.5±6.1	0.21
Urban residents (n, %)	210 (43.8)	310 (46.2)	0.43
Smoking (yes, n, %)	342 (71.4)	9 (1.3)	<0.001
Alcohol drinking (yes, n, %)	193 (40.3)	30 (4.5)	<0.001
Education (yr, n, %)			<0.001
0–6	221 (46.1)	374 (55.8)	
7–9	139 (29.0)	176 (26.2)	
≥10	119 (24.9)	121 (18.0)	
Physical activity (n, %)			0.07
Low	30 (6.3)	42 (6.3)	
Moderate	198 (41.3)	322(48.0)	
High	251 (52.4)	307 (45.8)	
Self-reported CVD‡ (n, %)	27 (5.6)	34 (5.1)	0.68
Family history of diabetes§ (n, %)	48 (10.0)	83(12.4)	0.35
Family history of CVD§ (n, %)	75 (15.7)	126(18.8)	0.25
Post-menopause (n, %)		593 (88.6)	
Waist circumference (cm)	83.34±10.39	80.00±9.87	<0.001
Hip circumference (cm)	90.98±5.79	91.90±6.58	0.03
Waist-to-hip ratio	0.91±0.07	0.87±0.07	<0.001
Total body fat mass (kg)	13.5±5.1	18.8±5.6	<0.001
Trunk fat mass (kg)	7.57±3.32	10.01±3.45	<0.001
Leg fat mass (kg)	3.64±1.35	5.56±1.73	<.0001
Ferritin (mg/l)||	149.8 (97.5–219.5)	111.6 (73.10–165.4)	<0.001

*Data are mean ± SD, median (interquartile range) or number (%); *P* value was calculated after adjusted for age and urban/rural residence (where appropriate).

‡ Self-reported CVD including stroke and coronary heart disease.

§ Parents or siblings had a history of diabetes or CVD.

|| This variable was log-transformed before analysis.

In Spearman correlation analyses, we found that plasma ferritin was positively correlated with waist circumference, waist-to-hip ratio, total body fat mass and trunk fat mass, while inversely correlated with leg fat mass in both men (r = 0.16, 0.26, 0.19, 0.22 and −0.12, respectively, all *P*<0.05) and women (r = 0.16, 0.16, 0.08, 0.17 and −0.12, respectively, all *P*<0.05) after adjusted for age and residence (Table 2). Multivariate linear regression analyses also showed that nature log-transformed ferritin levels were positively associated with trunk fat mass in both genders (β = 0.33 ± 0.08 for men and β = 0.21±0.05 for women, both *P*<0.001), and inversely associated with leg fat mass in women (β = −0.14 ± 0.05, *P* = 0.005). Ferritin levels also decreased with increased leg fat mass in men but did not reach the statistic significance (β = −0.12 ± 0.09, *P* = 0.15).

**Table 2:** Associations of plasma ferritin and indices of body fat distribution.

	Men (n = 479)				Women (n = 671)			
	Spearman correlation*		Standardized regression †		Spearman correlation*		Standardized regression†	
	r	*P*	β (SE)	*P*	r	*P*	β (SE)	*P*
Waist circumference‡	0.16	<0.001	0.27 (0.09)	0.003	0.16	<0.001	0.23 (0.06)	<0.001
Hip circumference‡	−0.02	0.71	−0.01 (0.09)	0.88	−0.10	0.007	−0.15 (0.06)	0.022
Waist-to-hip ratio	0.26	<0.001	0.23 (0.05)	<0.001	0.16	<0.001	0.14 (0.04)	<0.001
Total body fat	0.19	<0.001	0.22 (0.05)	<0.001	0.08	0.031	0.09 (0.04)	0.026
Trunk fat mass§	0.22	<0.001	0.33 (0.08)	<0.001	0.17	<0.001	0.21 (0.05)	<0.001
Leg fat mass§	−0.12	0.010	−0.12 (0.09)	0.15	−0.12	0.002	−0.14 (0.05)	0.005

*Adjusted for age and residence (urban/rural).

† Adjusted for age, residence (urban/rural), alcohol drinking, smoking, education attainment, physical activity, self-reported CVD, and family history of diabetes and CVD and menopause status (in women participants); plasma ferritin concentrations were nature log-transformed.

‡ Additionally adjusted for waist circumference or hip circumference (each other).

§ Additionally adjusted for trunk fat mass or leg fat mass (each other).

**Figure 1:**
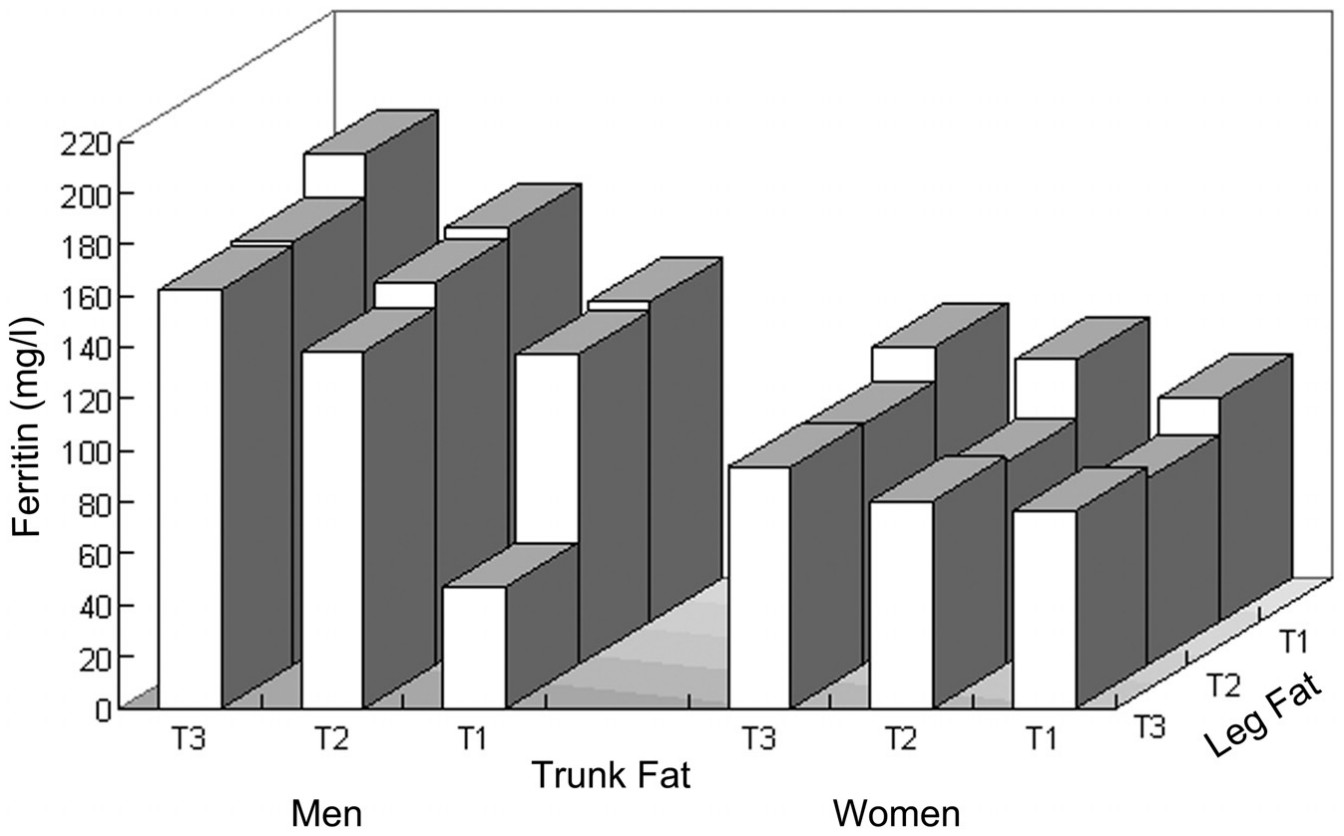
Adjusted mean levels of plasma ferritin according to the tertiles of trunk and leg fat mass. Data are geometric means for after adjusted for age, residence, alcohol drinking, smoking, educational attainment, physical activity, self-reported CVD, family history of diabetes and CVD and menopause status (in women participants).

As shown in Figure 1, after isolating 9 groups of participants with different fat distribution types according to the tertiles of trunk fat and leg fat mass, plasma ferritin levels trended to increase with more trunk fat mass both in men and women, as well as less leg fat mass in men. In women participants, ferritin levels were lower in bottom and middle tertiles than those in the highest tertile of leg fat mass, though they did not show a regular increased trend across the tertiles of leg fat mass.

## Discussion

This is the first study to report the opposite associations of trunk fat and leg fat depots with plasma ferritin concentrations. The positive association of ferrtin with waist circumference, waist-to-hip ratio, and trunk fat mass observed in current study was in line with the fact that most of previous studies have reported the significant association between elevated ferritin levels and abdominal obesity [1], [2], [6]. More interestingly, we found a novel association between larger leg fat and lower ferritin levels. To our knowledge, although the favorable effect of leg fat on metabolic disease risk has been recognized, no study has reported its effect on circulating ferritin concentrations. Notably, the opposite associations of trunk fat and leg fat with ferritin levels were independent of each other and larger leg fat mass appeared to have favorable association with ferritin among different levels of trunk fat mass.

Regulation of ferritin is complex, and a number of factors such as oxidative stress, inflammation, oncogenes, growth factors and other stimuli were implicated [4]. Excess fat may promote fatty acid oxidation and lead to oxidative stress [10], which has been shown to contribute to ferritin induction. Since lower body fat is less metabolically active [11], and thus less involved in oxidative metabolic processes than upper fat mass. Indeed, a previously study has found that waist and hip circumferences had opposite association with plasma ascorbic acid (a known antioxidant) concentrations [12]. These findings suggest that fatty acid oxidation may be implicated in the association between regional fat distribution and circulating ferritin levels. It is also possible that the opposite associations are mediated by inflammatory factors. Several previous studies have reported that increased c-reactive protein (CRP) and interleukin-6 levels were associated with larger trunk or abdominal fat [13]–[15], while a recent study found an association between increasing lower body fat and lower CRP levels [16]. Nevertheless, further studies are needed to clarify the underlying mechanism between ferritin and fat distribution.

The present study has some limitations. Due to no DXA scanner available in Beijing fieldwork, only the participants in Shanghai were invited to have body fat measured. Moreover, because the present study was conducted in middle-aged and older Chinese (aged 50 to 70 years), the results might not generalize to general populations and other ethnic groups. In addition, we did observe an inverse correlation between leg fat and ferritin, however, after multiple adjustment for various confounders, the association did not reach the statistic significance in men. It might attribute to the relative smaller sample size in men than in women. Further studies with larger sample sizes are needed to establish the association between ferritin and leg fat depot in men.

In conclusion, we found that trunk fat and leg fat have opposite associations with plasma ferritin levels. Considering the crucial role of ferritin in diabetes risk, our findings provided a new insight to potential roles of leg fat depot in ferritin-diabetes association.
